# Prevalence of Metabolic Syndrome and Its Risk Factors Influence on Microvascular Complications in Patients With Type 1 and Type 2 Diabetes Mellitus

**DOI:** 10.7759/cureus.55478

**Published:** 2024-03-04

**Authors:** Asad Riaz, Shoaib Asghar, Salman Shahid, Haider Tanvir, Muhammad Hamza Ejaz, Mamuna Akram

**Affiliations:** 1 Medicine, East Kent Hospital University Foundation Trust, Ashford, GBR; 2 Internal Medicine, Sheikh Zayed Medical College/Hospital, Rahim Yar Khan, PAK; 3 Medicine, Bedfordshire Hospitals NHS Foundation Trust, Bedford, GBR; 4 Internal Medicine, City Hospital, Multan, PAK; 5 Obstetrics and Gynaecology, Lincoln County Hospital, United Lincolnshire Hospitals NHS Trust (ULHT), Lincoln, GBR

**Keywords:** diabetic neuropathy (dn), diabetic retinopathy (dr), body mass index (bmi ), triglycerides (tg), hypertension (htn), high-density lipoproteins (hdl-c), diabetic kidney disease (dkd), type 2 diabetes mellitus (t2dm), type 1 diabetes mellitus (t1dm), metabolic syndrome (metsy)

## Abstract

Background: Diabetes mellitus (DM) long-term macrovascular and microvascular complications pose significant health risks and increase mortality. In DM patients, metabolic syndrome (MetSy) either precedes or coexists with the condition. Central obesity, poor glycemic control, hypertension, elevated triglycerides (TG), and low high-density lipoproteins (HDL-C) are the components of MetSy. The purpose of this study is to investigate related diabetic microvascular complications in type 1 DM (T1DM) by comparing them with type 2 DM (T2DM), determine potential risk factors, and estimate prevalence based on the diagnosis of MetSy.

Methodology: This study included 160 T1DM and 160 T2DM patients, totaling 320 DM patients. It was carried out from April 20, 2022, to September 31, 2023, at the Sheikh Zayed Hospital, Rahim Yar Khan, in the Outdoor Diabetic Clinic and Medicine Department. A unique questionnaire was utilized to gather socio-demographic, general, clinical, and laboratory data for the MetSy criteria set forth by the International Diabetes Federation (IDF). The blood pressure, BMI, and waist circumference (WC) were measured, while venous fasting blood was used to assess biochemical markers such as HDL-C, TG, and fasting blood sugar. The microvascular diabetes complications were identified using abdominal ultrasound, fundus ophthalmoscopy, and routine laboratory tests. We quantified and analyzed these variables individually for T1DM and T2DM patients with or without MetSy and compared them in the presence or absence of diabetes microvascular complications.

Results: MetSy prevalence was 25.62% (41, n=160) for T1DM and 60.62% (97, n=160) for T2DM, totaling 43.12%. Among T1DM patients with MetSy, the majority were married males, aged 36-49 years, with a BMI of 26.69±2.20 kg/m2 and a WC of 85.12±4.23, and 67.5% (108) patients had diabetes microvascular complications. Comparatively, in T2DM with MetSy, the majority were married females aged 50-59 years with a BMI of 29.79 ± 4.65 kg/m² and a large WC of 93.43±4.49, and 75% (123) patients had diabetes microvascular complications. Overall, this study noted significant p-values for hypertension, elevated TG, low HDL-c, high WC, obesity, female gender in T2DM, and above 36 years of age in both groups with MetSy. Diabetic retinopathy (DR) at 32.4% (p<0.001) was the most prevalent T1DM microvascular complication, followed by nephropathy (30.6%), neuropathy (DN) at 28.1%, and gastroparesis (DG) at 22.3%. Whereas in T2DM, the prevalence of DN was 36.3% (p<0.001), followed by DKD (29.3%), DG (28.9%), and DR (24.9%).

Conclusion: Nearly a quarter of T1DM patients had MetSy, with increasing percentages of overweight and obese patients who are more likely to have DR, DKD, or DN. MetSy affects two-thirds of T2DM patients, with married obese females aged 50-59 being more susceptible than males, who are more likely to suffer DN, DKD, or DG. Risk factors that contribute to the MetSy burden in T1DM and T2DM include hypertension, poor glycemic management, low HDL-C, high TG, and a higher BMI or WC. Increasing age, female gender in T2DM, longer diabetes duration, and co-morbid hypertension were independent predictors of microvascular complications. DR, DN, DKD, and gastroparesis are the most prevalent diabetic microvascular sequelae. The clinical management of diabetic patients with healthy lifestyle adaptations, good glycemic control, antihypertensives, and statins will contribute greatly to MetSy prevention.

## Introduction

Diabetes mellitus (DM), one of the most prevalent metabolic disorders, is a major global public health concern due to its long-term microvascular and macrovascular complications. According to the International Diabetes Federation (IDF), the global diabetes prevalence was estimated to be 10.5% in people aged 20-79 (537 million people), where 6.7 million people die every year from associated diabetic complications [1]. Although DM affects all organs, blindness, amputation, and renal failure contribute significantly to the social and financial burden of this disease in both T1DM and T2DM patients [2].

Metabolic syndrome (MetSy) is characterized by central obesity, hyperglycemia, hypertension, elevated triglycerides, and low high-density lipoproteins (HDL) [2,3]. MetSy precedes or coexists with diabetes in 70-80% of cases [2], and it has been related to a threefold increase in the risk of cardiovascular disease (CVD), other microvascular sequelae, and early death [3,4].

The prevalence of MetSy in T1DM and T2DM is growing quickly all around the world. Although the precise cause of MetSy remains unknown, specialists believe that insulin resistance and central obesity are significant causes. Chronic hyperglycemia, genetic predisposition, aging, a sedentary lifestyle with less physical activity, new eating behaviors, inflammation, and hormonal changes may all play a role, but the impact may differ by the ethnicity of the population under study [2,4-5].

The potential variables could be classified as socio-demographic factors (age, gender, marital status), behavioral factors (obesity, smoking, alcohol intake, physical activity, adherence to diet, BMI), and clinical factors (diabetes duration, family history, blood sugar control, type of therapy, comorbidities like hypertension and lipid disorders) [5,6]. Research suggests that age [2], gender, marital status [5], family history of diabetes [6-7], diabetes duration ≥ 5 years [2-4,7-10], insufficient glycemic control [5,10-11], no adherence to a healthy balanced diet [11], lack of exercise [12-13], overweight and obesity [9-14], mixed hypoglycemic therapies [15], type of insulin therapy [16], use of statins for any dyslipidemia [2,17], and hypertension [2-4, 9-11,16-19] are forecasting factors of complications from microvascular disease among T1DM and T2DM patients.

The pathological alterations in the microvasculature from the potential factors result in microvascular problems in vital organs like the kidneys (nephropathy), the eyes (retinopathy), the nervous system (neuropathy), and the autonomic gastrointestinal system (gastroparesis) [10-14]. Diabetic retinopathy (DR) is the primary cause of blindness in the population with diabetes; diabetic neuropathy (DN) causes foot ulcers and amputations; diabetic gastroparesis (DG) causes gastrointestinal and malabsorption; and diabetic nephropathy is the leading cause of chronic diabetic kidney disease (DKD) [14,15]. Therefore, the detection and identification of risk factors for microvascular complications is imperative and may prevent the progression of complications.

The goal of this study is to evaluate the underlying diabetic microvascular complications of T1DM and compare them with T2DM, identify significant risk variables, and determine prevalence based on the MetSy diagnosis.

## Materials and methods

Operational definitions

In line with the American Heart Association and the revised IDF [1] MetSy criteria, a person diagnosed with MetSy must match the following criteria: central obesity plus any two of the four factors mentioned.

Central obesity is presumed when BMI exceeds 30 kg/m². Men with a waist circumference (WC) of at least 94 cm and women with a WC of at least 80 cm are considered centrally obese.

In addition, any two of the four factors listed below. Blood pressure of 130/85 mmHg or above, or treatment of pre-existing hypertension. Triglyceride levels greater than or equal to 150 mg/dL (1.7 mmol/L) or specific treatment for lipid abnormalities. Reduced high-density lipoprotein cholesterol (HDL-C) to less than 40 mg/dL (1.03 mmol/L) for men and 50 mg/dL (1.29 mmol/L) for women, or specific therapy of the lipid alterations. Fasting plasma glucose (FPG) levels greater than or equal to 100 mg/dL (5.6 mmol/L) or a prior diagnosis of type 2 diabetes. An oral glucose tolerance test is advised at these levels.

BMI categories include normal weight (18.5-24.9 kg/m²), underweight (<18.5 kg/m²), overweight (25-30 kg/m²), and obese (>30 kg/m²) [1,2]. Good glycemic control is defined as fasting blood sugar (FBS) levels below 130 mg/dL, while FBS levels above 130 mg/dL are considered poor control [1-3]. Good physical activity is defined as moderate-intensity exercise for at least 150 minutes per week (three days), while less or no exercise is considered poor physical activity [1-3]. Microvascular consequences of DM include diabetic nephropathy (microalbuminuria or macroalbuminuria), diabetic gastroparesis, diabetic retinopathy, and peripheral neuropathy [1-4] in both previously and newly diagnosed cases. Microalbuminuria can be characterized as the excretion of 30 to 300 mg/dL of albumin protein in urine, while values greater than 300 mg/dL are classified as macroalbuminuria.

Study design

This prospective cohort study was carried out from April 20, 2022, to September 31, 2023, at the diabetes outpatient clinic and medicine department of Sheikh Zayed Hospital, Rahim Yar Khan, Pakistan.

Patients with T1DM and T2DM who met three of the five MetSy criteria were included in the study. Patients with newly diagnosed diabetes, age under 15 years, pregnant women, secondary diabetes, surgical histories, incomplete medical records, end-stage renal disease, diabetes foot infections or ulcerations, and other concomitant comorbidities were excluded from this study.

Data collection

After satisfying the ethical research board of Sheikh Zayed Medical College and Hospital (permission reference number 361/IRB/SZMC/SZH), 160 T1DM patients and 160 T2DM patients, for a total of 320 patients with DM who met the inclusion criteria, were chosen. Informed consent was obtained after outlining the study’s objectives. All patients were given a printed, customized questionnaire proforma and interviewed.

The socio-demographic variables were age, smoking history, alcohol consumption, gender, and marital status. Clinical features included family history, duration of diabetes, physical activity, dietary adherence, obesity, medications (OHD, insulin, antihypertensive, statin), concomitant risk factors, and diabetes microvascular complications in each category. A labeled piece of plastic tape was used to measure the waist circumference at the umbilicus level. BMI was calculated by dividing weight in kilograms by height in meters squared (kg/m2). A standard mercury manometer was used to measure blood pressure on the right arm while seated. Laboratory variables such as fasting blood glucose, HDL-C, and TG were measured from a blood sample and analyzed.

Fundus ophthalmoscopy was used to diagnose retinopathy (the presence of microaneurysms, cotton wool spots, venous beading, dot and blot hemorrhages, neovascularization, and vitreous hemorrhage). Neuropathy was assessed using a history and neurological examination, which included paresthesia, tingling sensations, numbness, loss of vibration, and joint position sensations. Gastroparesis was detected clinically by history and a questionnaire. Diagnosing nephropathy in DM was also based on symptoms involving swelling of the hands, feet, or eyes, frequent and urgent urination, measurement of blood pressure, urinalysis, and kidney ultrasonography.

Data analysis

Statistical analysis was conducted using IBM Corp.'s SPSS version 22 (Armonk, NY, USA), utilizing the questionnaire proforma. For the qualitative variables, frequencies were assessed. The presence or absence of diabetes microvascular complications was compared between the MetSy+ and MetSy− groups of T1DM and T2DM using characteristic socio-demographic, clinical, and laboratory variables such as age, gender, marital status, waist circumference, BMI, obesity, physical activity, dietary habits, glycemic control, diabetes duration, treatment types, fasting plasma glucose, triglycerides, high-density lipoproteins, blood pressure, and history of comorbidities. The prevalence of microvascular complications of diabetes was calculated based on the presence or absence of MetSy in T1DM, T2DM, and overall DM patients. A p-value less than 0.05 was considered statistically significant.

## Results

This study included 160 T1DM patients and 160 T2DM patients, for a total of 320 participants with DM. The prevalence of MetSy was 25.62% (41, n=160) for T1DM and 60.62% (97, n=160) for patients with T2DM. Altogether, 138 (n=320) diabetic individuals (60 male and 78 female) had MetSy according to the IDF criteria, with an overall prevalence of 43.12%.

Patients were classified into groups based on their type of DM and whether or not they matched the various MetSy criteria. The diabetes microvascular complications were investigated for every characteristic of all the studied diabetic patients. Out of 160 T1DM participants, 25% (40) had higher fasting plasma glucose, 25.62% (41) had central obesity, 25.62% (41) had hypertension, 23.12% (37) had increased triglycerides (TG), and 23.75% (38) had low high-density cholesterol lipoproteins (HDL-C), for a total of 25.62% (41) individuals overall with MetSy. And among 160 T2DM patients, 57.5% (92) had higher fasting plasma glucose, 59.37% (95) had central obesity, 58.12% (93) had hypertension, 58.75% (94) had elevated triglycerides, and 49.37% (79) had low HDL-C, resulting in 60.62% (97) individuals having MetSy.

**Figure 1:**
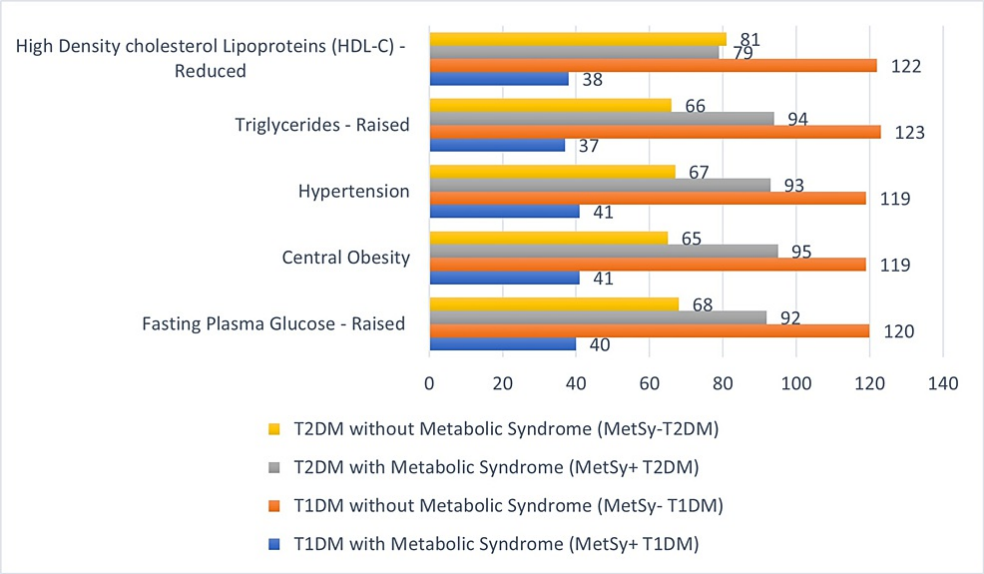
The prevalence of each component of the metabolic syndrome in the whole group of T1DM and T2DM patients (n=320)

Figure 1 displays the prevalence of each component of MetSy in the whole group of T1DM and T2DM patients.

Among T1DM with MetSy (41, n=160), the mean age of participants was 36 years, with a predominance of 36-49 year-olds (31.87%), predominantly males (25, 15.62%), and married patients. The average BMI for males was 26.69 ± 2.20 kg/m², with a MetSy prevalence of 15.62% (25), whereas the average BMI for females was 27.49 ± 1.66 kg/m², with a 10% prevalence (16). The prevalence of overweight was objectified at 14.37% (23), and 6.25% (10) had obesity. A large waist circumference (LWC) of 89.43 ± 4.65 was found in females as compared to males at 85.12 ± 4.23, with MetSy common in 25 (15.62%) males. The 67.5% (108, n=160) patients had diabetes-related microvascular complications. In T1DM, this study noted significant p-values for above-36-year-old age groups (p-value = 0.04), high WC in males (p-value = 0.04), higher WC in females (p-value = 0.03), BMI (p-value = 0.05), overweight (p-value = 0.05), and obese patients (p-value = 0.03).

Comparatively, in T2DM patients with MetSy (97, n=160), the mean age of participants was 52 years, with a predominance of the 50-59 age group (36.87%), predominantly in married (94, 58.75%) females (62, 38.57%). The average BMI for males was 28.61 ± 4.02 kg/m², with a MetSy prevalence of 21.87% (35), whereas the BMI for females was 29.79 ± 4.65 kg/m², with a 38.75% prevalence (62); the prevalence of overweight was objectified at 41.87% (67), and 14.37% (23) had obesity. A large WC of 93.43 ± 4.49 was found in females as compared to males at 91.53 ± 4.68, with MetSy common in 38.75% (62) females. The 75% (123, n=160) patients had diabetes-related microvascular complications. In T2DM, this study noted significant p-values for above-36-year-old age groups (p-value = 0.03), female gender (p-value = 0.04), high WC in males (p-value = 0.03), higher WC in females (p-value = 0.02), BMI in males (p-value = 0.04), BMI in females (p-value = 0.03), overweight (p-value = 0.03), and obese (p-value = 0.01) patients. Smoking and alcohol intake had no significant impact on the studied diabetic population, with insignificant p-values of 0.07 and 0.09.

Table 1 shows the socio-demographic and general characteristic features of these variables in the studied T1DM and T2DM populations with or without MetSy.

**Table 1:** Socio-demographic and general features of studied type 1 and type 2 diabetes mellitus participants (n=320)

Variables	Number (%)			Metabolic syndrome in type 1 diabetes mellitus (MetSy-T1DM), n=160			Metabolic syndrome in type 2 diabetes mellitus (MetSy-T2DM), n=160			Diabetes: microvascular complications			
	T1DM n=160	T2DM n=160	DM overall: n=320	MetSy+ 41 (25.62%)	MetSy– 119 (74.38%)	P-value	MetSy+ 97 (60.62%)	MetSy– 63 (39.38%)	P-value	Yes		Yes	No
										T1DM 108 (67.5%)	T2DM 123 (75%)	Overall DM 231 (72.19%)	Overall DM: 89 (27.81%)
Age													
15-35 (years)	98 (61.25%)	25 (15.62%)	123 (38.44%)	15 (9.37%)	83 (69.17%)	0.07	02 (1.25%)	23 (14.37%)	0.09	59 (36.87%)	06 (3.75%)	65 (20.31%)	58 (18.12%)
36-49 (years)	51 (31.87%)	54 (33.75%)	105 (32.81%)	18 (11.25%)	33 (20.62%)	0.04	36 (22.5%)	18 (11.25%)	0.03	39 (24.37%)	43 (26.87%)	82 (25.62%)	23 (7.18%)
50-59 (years)	11 (6.87%)	81 (50.62%)	92 (28.75%)	8 (5%)	3 (1.87%)	0.04	59 (36.87%)	22 (13.75%)	0.03	10 (6.25%)	74 (46.25%)	84 (26.25%)	08 (2.5%)
Smoking													
Yes	26 (16.25%)	43 (26.87%)	69 (21.56%)	16 (10%)	10 (6.25%)	0.07	24 (15%)	19 (11.87%)	0.07	24 (15%)	38 (23.75%)	62 (19.37%)	07 (2.18%)
No	134 (83.75%)	117 (73.12%)	251 (78.44%)	25 (15.62%)	109 (68.12%)	0.13	73 (45.62%)	44 (27.5%)	0.08	84 (52.5%)	83 (51.87%)	167 (52.18%)	84 (26.25%)
Alcohol													
Yes	02 (1.25%)	14 (8.75%)	16 (05%)	1 (0.62%)	1 (0.62%)	0.09	12 (7.5%)	2 (1.25%)	0.09	02 (1.25%)	13 (8.12%)	15 (4.68%)	01 (0.31%)
No	158 (98.75%	146 (91.87%)	304 (95%)	40 (25%)	118 (73.75%)	0.12	85 (53.12%)	61 (38.12%)	0.09	106 (66.25%)	110 (68.75%)	216 (67.5%)	88 (27.5%)
Gender													
Male	95 (59.37%)	77 (48.12%)	172 (53.75%)	25 (15.62%)	70 (43.75%)	0.08	35 (21.87%)	42 (26.25%)	0.09	61 (38.12%)	55 (34.37%)	116 (36.25%)	56 (17.5%)
Female	65 (40.62%)	83 (51.87%)	148 (46.25%)	16 (10%)	49 (30.62%)	0.09	62 (38.75%)	21 (13.12%)	0.04	47 (29.37%)	68 (42.5%)	115 (35.93%)	33 (10.31%)
Marital status													
Married	116 (72.5%)	156 (97.5%)	272 (85%)	29 (18.12%)	87 (54.37%)	0.10	94 (58.75%)	62 (38.75%)	0.06	101 (63.12%)	121 (75.62%)	222 (69.37%)	50 (15.62%)
Unmarried	44 (27.5%)	04 (2.5%)	48 (15%)	12 (7.5%)	32 (20%)	0.09	3 (1.87%)	1 (0.62%)	0.06	07 (4.37%)	02 (1.25%)	09 (2.81%)	39 (12.18%)
Waist circumference													
Male (cm)	85.12 ± 4.23	91.53 ± 4.68	89.63 ± 6.58	25 (15.62%)	70 (43.75%)	0.04	35 (21.87%)	42 (26.25%)	0.03	61 (38.12%)	55 (34.37%)	116 (36.25%)	56 (17.5%)
Female (cm)	89.43 ± 4.65	93.43 ± 4.49	91.35 ± 6.57	16 (10%)	49 (30.62%)	0.03	62 (38.75%)	21 (13.12%)	0.02	47 (29.37%)	68 (4.25%)	115 (35.93%)	33 (10.31%)
BMI													
Male (kg/m²)	26.69 ± 2.20	28.61 ± 4.02	28.89 ± 3.74	25 (15.62%)	70 (43.75%)	0.05	35 (21.87%)	42 (26.25%)	0.04	61 (38.12%)	55 (34.37%)	116 (36.25%)	56 (17.5%)
Female (kg/m²)	27.49 ± 1.66	29.79 ± 4.65	29.15 ± 5.29	16 (10%)	49 (30.62%)	0.05	62 (38.75%)	21 (13.12%)	0.03	47 (29.37%)	68 (42.5%)	115 (35.93%)	33 (10.31%)
Obesity													
Yes, BMI > 30 kg/m²	12 (7.5%)	32 (20%)	44 (13.75%)	10 (6.25%)	02 (1.25%)	0.03	23 (14.37%)	09 (5.62%)	0.01	12 (7.5%)	31 (19.37%)	43 (13.43%)	01 (0.31%)
Overweight, BMI 25-30 kg/m²	106 (66.25%)	117 (73.12%)	223 (69.69%)	23 (14.37%)	83 (51.87%)	0.05	67 (41.87%)	50 (31.25%)	0.03	81 (50.62%)	79 (49.37%)	160 (50%)	63 (19.68%)
No; BMI < 25	42 (26.25%)	11 (6.8%)	53 (16.56%)	08 (05%)	34 (21.25%)	0.09	07 (4.37%)	04 (2.5%)	0.07	15 (9.37%)	13 (8.12%)	28 (8.75%)	25 (7.81%)

In this study, 86.87% (139, n = 160) of patients with T1DM had a family history of diabetes, and 88.75% (142) had the disease for more than five years. Insulin was the preferred medication for 98.13% (157) T1DM participants to manage glycemic control; 52.5% (84) used antihypertensive medication; and 53.75% (86) used statins to treat low HDL-C or high TG levels. The majority of these 93 (58.12%, n160) showed low physical activity, either not exercising at all or performing less than 150 minutes per week. 73.75% (118) had central obesity, 59.37% (95) did not follow a healthy sugar-free diet, and 89.38% (143) had poor glycemic control with fasting blood sugar levels higher than 130 mg/dL. The most prevalent MetSy problems in type 1 diabetics were high blood pressure (hypertension) in 52.5% (84, p-value = 0.04), low HDL-C in 53.75% (86, p-value = 0.03), and high TG in 41.87% (67, p-value = 0.02).

In comparison with T2DM, 70% (112, n = 160) patients had a family history of diabetes, whereas 80.63% (129) had the disease for more than 5 years. For glycemic management, 36.87% (59) of T2DM patients used oral hypoglycemic medications (OHD), 40% (64) combined OHD with insulin, and 23.13% (37) used insulin only. Whereas, 72.5% (116) used antihypertensive medication, and 65.62% (105) used statins to treat low HDL-C or high TG levels. Most of these 66.25% (106) had poor physical health, 74.37% (119) had no adherence to a balanced diet, and 93.12% (149) were centrally obese. The most common MetSy issues in type 2 diabetics were high blood pressure (hypertension) in 72.5% (116, p-value = 0.02), low HDL-C in 65.62% (105, p-value = 0.002), and high TG in 80.63% (129, p-value = 0.004).

The study revealed a significant p-value < 0.05 for hypertension, abdominal obesity, poor glycemic control, hypertriglyceridemia, and low HDL (all components of MetSy) in both T1DM and T2DM (Table 2).

**Table 2:** Clinical and laboratory variables of studied type 1 and type 2 diabetes mellitus participants (n=320) Good*: perform at least 150 mins/week (3 days) of moderate-intensity exercise Poor*: perform less than 150 mins/week or no exercise at all HDL-C: high-density lipoprotein, TG: triglycerides

Variables	Number (%)			Metabolic syndrome in type 1 diabetes mellitus (MetSy-T1DM), n=160			Metabolic syndrome in type 2 diabetes mellitus (MetSy-T2DM), n=160			Diabetes: microvascular complications			
	T1DM n=160	T2DM n=160	DM Overall n=320	MetSy+ 41 (25.62%)	MetSy– 119 (74.38%)	P-value	MetSy+ 97 (60.62%)	MetSy– 63 (39.38%)	P-value	Yes		Yes	No
										T1DM 108 (67.5%)	T2DM 123 (75%)	Overall DM 231 (72.19%)	Overall DM 89 (27.81%)
Family history of diabetes													
Yes	139 (86.87%)	112 (70%)	251 (78.43%)	37 (23.12%)	102 (63.75%)	0.08	93 (58.12%)	19 (77.88%)	0.06	97 (60.62%)	102 (63.75%)	199 (62.18%)	52 (16.25%)
No	21 (13.13%)	48 (30%)	69 (21.56%)	04 (2.5%)	17 (10.63%)	0.12	04 (2.5%)	44 (27.5%)	0.09	11 (6.87%)	21 (13.13%)	32 (10%)	37 (11.56%)
Diabetes duration													
≥5 years	142 (88.75%)	129 (80.63%)	271 (84.68%)	38 (23.75%)	104 (65%)	0.05	91 (56.87%)	38 (23.75%)	0.03	101 (63.12%)	103 (64.37%)	204 (63.75%)	67 (20.93%)
<5 years	18 (11.25%)	31 (19.37%)	49 (15.31%)	03 (1.87%)	15 (9.38%)	0.13	06 (3.75%)	25 (15.63%)	0.12	07 (4.38%)	20 (12.5%)	27 (8.43%)	22 (6.87%)
Medications													
Oral only	00 (0%)	59 (36.87%)	59 (18.43%)	00 (0%)	00 (0%)	0.50	23 (14.38%)	36 (22.5%)	0.09	00 (0%)	36 (22.5%)	36 (11.25%)	23 (7.18%)
Oral + insulin	03 (1.87%)	64 (40%)	67 (20.93%)	03 (1.87%)	00 (0%)	0.01	48 (30%)	16 (10%)	0.04	03 (1.87%)	58 (36.25%)	61 (19.06%)	06 (1.87%)
Insulin only	157 (98.13%)	37 (23.13%)	194 (60.62%)	38 (23.75%)	119 (74.37%)	0.08	26 (16.25%)	11 (6.87%)	0.06	105 (65.63%)	29 (18.13%)	134 (41.87%)	60 (18.75%)
Antihypertensive	84 (52.5%)	116 (72.5%)	200 (62.5%)	41 (25.62%)	43 (26.88%)	0.04	93 (58.12%)	23 (14.37%)	0.02	74 (46.25%)	98 (61.25%)	172 (53.75%)	28 (7.81%)
Statins	86 (53.75%)	105 (65.62%)	191 (59.68%)	41 (25.62%)	45 (28.12%)	0.04	97 (60.62%)	08 (5.0%)	0.03	81 (50.63%)	106 (66.25%)	187 (58.43%)	04 (1.25%)
Physical activity													
Good*	67 (41.87%)	54 (33.75%)	121 (37.81%)	02 (1.25%)	65 (40.62%)	0.32	21 (13.13%)	33 (20.62%)	0.15	17 (10.63%)	19 (11.87%)	36 (11.25%)	85 (26.56%)
Poor*	93 (58.12%)	106 (66.25%)	199 (62.18%)	39 (24.38%)	54 (33.75%)	0.05	76 (47.5%)	30 (18.75%)	0.04	91 (56.87%)	104 (65%)	195 (60.93%)	04 (1.25%)
Adherence to diet													
Yes	65 (40.62%)	41 (25.62%)	106 (33.12%)	05 (3.13%)	60 (37.5%)	0.15	24 (15%)	17 (10.63%)	0.09	20 (12.5%)	14 (87.5%)	34 (10.62%)	72 (22.5%)
No	95 (59.37%)	119 (74.37%)	214 (66.87%)	36 (22.5%)	59 (36.87%)	0.08	73 (45.62%)	46 (28.75%)	0.06	88 (55%)	109 (68.13%)	197 (61.56%)	17 (5.31%)
Obesity													
Yes	118 (73.75%)	149 (93.12%)	267 (83.43%)	41 (25.62%)	77 (48.13%)	0.03	95 (59.37%)	54 (33.75%)	0.01	108 (67.5%)	123 (76.88%)	231 (72.18%)	36 (11.25%)
No	42 (26.25%)	11 (6.88%)	53 (16.56%)	00 (0%)	42 (26.25%)	0.50	02 (1.25%)	09 (5.63%)	0.40	00 (0%)	00 (0%)	00 (0%)	53 (16.56%)
Glycemic control (FBS <130 mg/dl)													
Good	17 (10.62%)	12 (7.5%)	29 (9.06%)	01 (0.63%)	16 (10%)	0.08	05 (3.13%)	07 (43.75%)	0.09	15 (9.37%)	03 (1.88%)	18 (5.62%)	11 (3.43%)
Poor	143 (89.38%)	148 (92.5%)	291 (90.93%)	40 (25%)	103 (64.37%)	0.04	92 (57.5%)	56 (35%)	0.003	93 (58.12%)	120 (75%)	213 (66.56%)	78 (24.37%)
Hypertension													
Yes	84 (52.5%)	116 (72.5%)	200 (62.5%)	41 (25.62%)	43 (26.88%)	0.04	93 (58.12%)	23 (14.38%)	0.02	74 (46.25%)	98 (61.25%)	172 (53.75%)	28 (8.75%)
No	76 (47.5%)	44 (27.5%)	120 (37.5%)	00 (0%)	76 (47.5%)	0.50	04 (2.5%)	40 (25%)	0.40	34 (21.25%)	25 (15.62%)	59 (18.43%)	61 (19.06%)
HDL-C													
Low	86 (53.75%)	105 (65.62%)	191 (59.68%)	38 (23.75%)	48 (30%)	0.03	79 (49.37%)	26 (16.25%)	0.002	75 (46.87%)	87 (54.38%)	162 (50.62%)	29 (90.62%)
High	74 (46.25%)	55 (34.38%)	129 (40.31%)	03 (1.87%)	71 (44.38%)	0.12	18 (11.25%)	37 (23.13%)	0.09	33 (20.62%)	36 (22.5%)	69 (21.56%)	60 (18.75%)
TG													
Low	93 (58.12%)	31 (19.38%)	124 (38.75%)	04 (2.5%)	89 (55.63%)	0.13	03 (1.87%)	28 (17.5%)	0.10	27 (16.87%)	17 (10.63%)	44 (13.75%)	80 (25%)
High	67 (41.87%)	129 (80.63%)	196 (61.25%)	37 (23.12%)	30 (18.75%)	0.02	94 (58.75%)	35 (21.88%)	0.004	81 (50.62%)	106 (66.25%)	187 (58.43%)	09 (2.81%)
Components of metabolic syndrome (3 or more)	41 (25.62%)	97 (60.62%)	138 (43.12%)	41 (25.62%)	119 (74.38%)	0.001	97 (60.62%)	63 (39.38%)	0.001	41 (25.62%)	97 (60.63%)	138 (43.12%)	182 (56.87%)

In this study, the prevalence of MetSy in T1DM and T2DM was 25.62% (41, n = 160) and 60.62% (97, n = 160), with significant p-values of <0.001 for all diabetic microvascular complications (Table 3).

**Table 3:** Diabetic microvascular complications prevalence in studied type 1 and type 2 diabetes mellitus participants with or without metabolic syndrome (n=320)

Diabetic microvascular complications	Prevalence (%)			Metabolic syndrome in type 1 diabetes mellitus (MetSy-T1DM), n=160			Metabolic syndrome in type 2 diabetes mellitus (MetSy-T2DM), n=160		
	T1DM n=160	T2DM n=160	DM overall n=320	MetSy+ 41 (25.62%)	MetSy– 119 (74.38%)	P-value	MetSy+ 97 (60.62%)	MetSy– 63 (39.38%)	P-value
Diabetic neuropathy	45 (28.1%)	58 (36.3%)	103 (32.19%)	36 (22.5%)	09 (5.62%)	0.01	43 (26.87%)	15 (9.37%)	<0.001
Diabetic nephropathy	49 (30.6%)	47 (29.3%)	96 (30%)	38 (23.75%)	11 (6.8%)	0.02	40 (24.9%)	07 (4.37%)	<0.001
Microalbuminuria	34 (21.3%)	36 (22.5%)	70 (21.87%)	27 (16.87%)	07 (4.37%)	0.012	31 (19.37%)	05 (3.12%)	<0.001
Macroalbuminuria	15 (8.8%)	11 (6.8%)	26 (8.12%)	10 (6.25%)	05 (3.12%)	0.012	10 (6.25%)	01 (0.62%)	<0.001
Diabetic retinopathy	52 (32.4%)	40 (24.9%)	92 (28.75%)	38 (23.75%)	14 (8.75%)	0.01	36 (22.5%)	04 (2.5%)	<0.001
Diabetic gastroparesis	36 (22.3%)	46 (28.9%)	82 (25.62%)	24 (15%)	12 (7.5%)	0.02	41 (25.62%)	05 (3.12%)	<0.001

**Figure 2:**
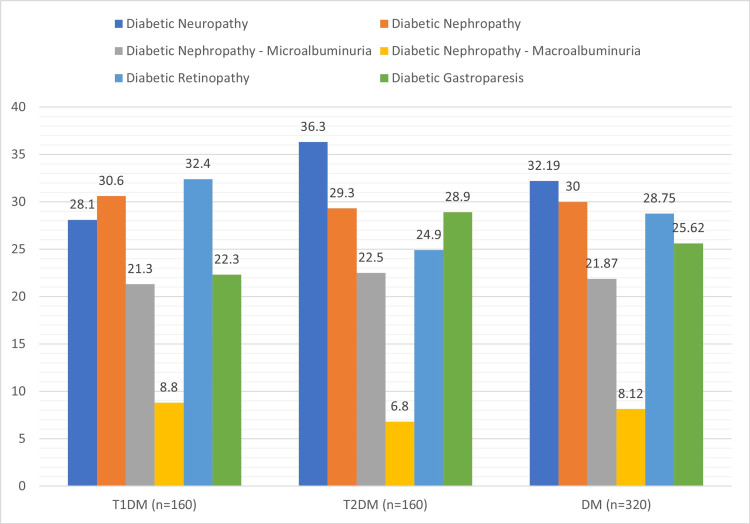
Prevalence of diabetic microvascular complications in T1DM, T2DM, and overall DM

Retinopathy was the most prevalent T1DM microvascular complication, accounting for 32.4%, followed by nephropathy (30.6%), which included microalbuminuria (21.3%), neuropathy (28.1%), and gastroparesis (22.3%). The most common T2DM microvascular complication was neuropathy (36.3%), followed by nephropathy (29.3%), with mainly microalbuminuria (29.3%), gastroparesis (28.9%), and retinopathy (24.9%) (Figure 2).

## Discussion

MetSy prevalence varies between studies. The IDF and AHA agreed that in addition to central obesity, the presence of two of the four risk variables (hypertension, elevated TG, low HDL-C, and poor glycemic control) established a diagnosis of MetSy [1-3]. MetSy tripled the risk of cardiovascular disease, various microvascular sequelae, and increased morbidity and mortality [3-4,8], particularly in patients with MetSy who also have diabetes [8-11]. The available research can provide a realistic estimate of the increasing prevalence of MetSy in diabetic patients. The purpose of this study was to determine the prevalence of T1DM and T2DM with or without MetSy, as well as the various characteristics and diabetic microvascular complications among its participants, based on the MetSy criteria.

This study displayed that the prevalence of MetSy in this population was 25.62% for T1DM and 60.62% for patients with T2DM, an overall prevalence of 43.12% according to the revised IDF definition. Lee et al. reported comparable increasing MetSy numbers in 2019 among T1DM at 54.9% [12], a study by Chillarón et al. at 31.9% [13], Udell et al. at 25.5% [14], and Chillarón et al. at 40% [15]. The result obtained for T2DM with MetSy concurs with those reported by Asghar et al. at 65% [2], Nsiah et al. at 58% [8], and Abagre et al. at 68.8% [16]. A higher prevalence of 71.9% was observed in a study by Bhatti et al. [17] and 73.3% by Raman et al. [18]. However, the prevalence was higher than that reported by Dündar and Akıncı at 43.8% [19] and Chen et al. at 51.4% [20]. These variations in MetSy prevalence can be explained by factors such as MetSy criteria, time of research, population ethnicity, and sociodemographic disparities [4,6,20-21]

Among T1DM with MetSy (41, n160), the mean age of participants was 36 years with a predominance of 36-49 years age group (31.87%), predominantly in males 25 (15.62%) as compared to females (10%) and majority as married patients (18.11%), as reported by Asghar et al. [2] in Pakistan, Bhatti et al. [17] in India, and Vest et al. [21]. The average BMI for males was 24.69 ± 3.93 kg/m², with a MetSy prevalence of 15.62% (25, n = 41), whereas the BMI for females was 26.49 ± 2.95 kg/m², with a 10% prevalence, and the prevalence of overweight was objectified at 14.37% (23) and 6.25% (10) had obesity. A LWC of 83.43 ± 3.67 was found in females as compared to males at 82.12 ± 3.23, with MetSy common in 15.62% of males. The 67.5% (108, n = 160) patients had diabetes-related microvascular complications. These findings are similar to those reported by Fawwad et al. in Balochistan, Pakistan [22], and by Khanam et al. [23] in Bangladesh.

Comparatively, in T2DM patients with MetSy (97, n160), the mean age of participants was 52 years, with a predominance of 50-59 years of age (36.87%), predominantly in married 94 (58.75%) and females 62 (38.57%), as studied by Asghar et al. in Pakistan [2] and Li X et al. [24] in the studied population. The average BMI for males was 28.61 ± 2.02 kg/m², with a MetSy prevalence of 21.87% (35, n = 97), whereas the BMI for females was 29.49 ± 2.65 kg/m², with a 38.75% prevalence (62), and the prevalence of overweight was objectified at 41.87% (67), and 14.37% (23) had obesity. A LWC of 93.43 ± 4.49 was found in females as compared to males at 89.53 ± 3.68, with MetSy common in 62 (38) females. The 75% (123, n = 160) patients had diabetes-related microvascular complications. These results are in line with those reported by Raman et al. [18] and Dundar and Akinci 2022 from Turkey [19].

In this study, 86.87% (139) of T1DM patients had a family history of diabetes, and 88.75% (142) had the disease for more than five years. Insulin was the preferred medication for 98.13% (157) T1DM participants to manage glycemic control; 52.5% (84) used antihypertensive medication; and 53.75% (86) used statins to treat low HDL-C or high TG levels. The majority of these 58.12% (93, n = 160) showed low physical activity, either not exercising at all or performing less than 150 minutes per week. 73.75% (118) had central obesity, 59.37% (95) did not follow a healthy sugar-free diet, and 89.38% (143) had poor glycemic control with fasting blood sugar levels higher than 130 mg/dL. The most prevalent MetSy problems in type 1 diabetics were high blood pressure (hypertension) in 25.62% (41), low HDL-C in 23.75% (38), and high TG in 23.12% (37).

In comparison with T2DM, 70% (112, n = 160) patients had a family history of diabetes, whereas 80.63% (129) had the disease for more than five years. For glycemic management, 36.87% (59) of T2DM patients used oral hypoglycemic medications (OHD), 40% (64) combined OHD with insulin, and 23.13% (37) used insulin only. Whereas, 72.5% (116) used antihypertensive medication, and 65.62% (105) used statins to treat low HDL-C or high TG levels. Most of these 66.25% (106) had poor physical health, 74.37% (119) had no adherence to a balanced diet, and 93.12% (149) were centrally obese. The most common MetSy issues in type 2 diabetics were high TG in 58.75% (94), high blood pressure (hypertension) in 58.12% (93), and low HDL-C in 49.37% (79).

Hypertension was the most common co-morbidity seen in this study, with an overall 62.5% (200, n320), compared to 52.25% (84, n160) in T1DM and 72.5% (116) in T2DM. Studies by Asghar et al. [2], Seid Ma et al. [11], Abagre et al. [16], Maloberti et al. [25], and Taliti et al. [26] found that hypertension was the most determinant factor for MetSy prevalence. In an Indian study conducted by Bhatti et al. [17], hypertension was reported in 82% of the Asian population.

However, Raman et al. [18] and Dundar and Akinci 2022 from Turkey [19] found that visceral obesity was the most prevalent criterion (68.3%). A study by Chen et al. argued that only low HDL-C and elevated fasting blood glucose were associated with all-cause and cardiovascular mortality [20]. Overall, this study noted significant p-values for hypertension, abdominal obesity, poor glycemic control, elevated TG, low HDL-c, high waist circumference, obesity, female gender in T2DM, and above 36 years of age in both groups with MetSy.

Retinopathy (32.4%, p-value <0.001) was the most prevalent T1DM microvascular complication, followed by nephropathy (30.6%), neuropathy (28.1%), and gastroparesis (22.3%). Diabetic nephropathy was found overall in T1DM at 23.75% (38, n = 160) and T2DM at 24.9% (40, n = 160), which is similar to that reported by Khanam et al. [23] at 21.3%. Microalbuminuria was observed in 21.87% (70, n = 320), while macroalbuminuria was found in 8.12% (26, n = 320). The studies of Huang et al. [27] and Hsu et al. [28] reported that patients with MetSy were more prone to develop diabetic retinopathy and diabetic kidney disease.

Whereas, in T2DM, the prevalence of neuropathy was 36.3% (p-value <0.001), followed by nephropathy (29.3%), gastroparesis (28.9%), and retinopathy (24.9%). Diabetic neuropathy was noted at a prevalence of 22.5% in T1DM and 26.87% in T2DM patients with MetSy, with an overall percentage of 28.1% (45, n = 320), which is comparable to studies by Asghar et al. at 10.8% [2] and Khanam et al. at 16.8% [23]. MetSy significantly increases the prevalence of diabetic microvascular problems among T1DM and T2DM individuals (p-value <0.001) [27-29].

The goal should be to transition from sedentary to active lifestyles by adhering to a balanced diet and increasing physical exercise, educating on the consumption of quality food (cutting excess calories), and lowering excess weight, particularly abdominal girth. Correcting these metabolic problems is critical for reducing the disease's influence on the microvascular system [28-30].

This study exempted patients with newly diagnosed diabetes, those under the age of 15, pregnant women, secondary diabetes, surgical history, insufficient medical records, end-stage renal disease, diabetes foot infections or ulcerations, and other concomitant comorbidities. Nonetheless, this study was capable of estimating several variables in a single trial.

## Conclusions

Nearly a quarter of T1DM patients had metabolic syndrome, with increasing percentages of overweight and obese patients who are more likely to have retinopathy, diabetic kidney disease, or neuropathy. MetSy affects two-thirds of T2DM patients, with married obese females aged 50-59 being more susceptible than males, who are more likely to develop diabetic neuropathy, DKD, or gastroparesis. Risk factors that contribute to the MetSy burden in T1DM and T2DM include hypertension, poor glycemic management, low HDL-C, high TG, and a higher BMI or WC. Increasing age, female gender in T2DM, longer uncontrolled diabetes duration, and co-morbid hypertension were independent predictors of microvascular complications.

Diabetic retinopathy, neuropathy, nephropathy, and gastroparesis are the most prevalent microvascular complications in both T1DM and T2DM; immediate attention is needed to stop further detrimental diabetic macrovascular complications such as cerebrovascular accidents, cardiovascular diseases, blindness, or end-stage renal disease. The clinical management of diabetic patients with healthy lifestyle modifications, better glycemic control, antihypertensives, and statins will significantly contribute to MetSy prevention and diabetes microvascular complications.
